# Role of myosin Va on HTLV-1 p8I protein's traffic to cell surface

**DOI:** 10.1186/1742-4690-12-S1-P1

**Published:** 2015-08-28

**Authors:** Jaqueline G Jesus, Rui MPS Júnior, Maria F de Castro-Amarante, Simone K Haddad, Enilza M Espreáfico, Genoveffa Franchini, Luiz CJ Alcântara

**Affiliations:** 1 Gonçalo Moniz Research Center, Oswaldo Cruz Foundation, R. Waldemar Falcão, 121, Candeal Salvador, Bahia, 40296-710, Brazil; 2 Faculty of Medicine of Ribeirão Preto, University of São Paulo – Av. Bandeirantes, 3900, Monte Alegre – Ribeirão Preto, São Paulov 14049-900, Brazil; 3 Animal Models and Retroviral Vaccines Section, National Cancer Institute, National Institute of Health, Bethesda, USA; 4 Faculty of Pharmaceutics of Ribeirão Preto, University of São Paulo – Av. do Café, s/nº, Ribeirão Preto, São Paulo, 14040-903, Brazil; 5 Blood Center of Ribeirão Preto – R. Tenente Catão Roxo, 2501, Ribeirão Preto, São Paulo, 14051-140, Brazil

## 

HTLV-1 ORF-I encodes the 99 amino acid p12 protein which can be proteolytically cleaved at the amino terminus to generate the p8 protein. The first proteolytic cleavage removes the ER retention/retrieval signal at the amino terminus of p12, while the second cleavage generates the p8 protein. The p12 protein localizes to cellular endomembranes, within the ER and Golgi apparatus, while p8 traffics to lipid rafts at the cell surface and is recruited to the immunological synapse upon T-cell receptor (TCR) ligation. In secretory vesicle transport, vesicles produced as post-Golgi are moved along the cytoskeleton by motor proteins. The unconventional myosin motor, myosin V, moves several cargoes including secretory vesicles, synaptic vesicles, and the endoplasmic reticulum. To understand if p8 traffics from Golgi apparatus to cell surface on a myosin Va dependent manner, Jurkat T cells were transfected with pMEp12 plasmids which express variants (p12WT, p12Δ29 and p12G29S) of the fusion protein of HTLV-1 p12I tagged with the influenza hemagglutinin (HA1) tag and with the Myo Va full-tail neuronal-eGFP conjugated (MyoVa FTNeu-eGFP) plasmid which expresses a negative dominant of myosin Va and competes for intracellular ligands with cellular putative myosin. Both p12/p8 and myosin Va proteins intracellular localization were analyzed by indirect immunofluorescence assay using antibodies against the HA-tag and the Myo Va protein. Confocal microscopy and image collection was performed by using a p12I/p8I proteins Zeiss LSM 780 microscope (Carl Zeiss Optical, Chester, Va.) with Adobe Photoshop CC software. It was reported p12I expression in Jurkat T as previous described was shown in perinuclear region which might be RE and Golgi apparatus and that p12I/p8I and MyoVa proteins colocalizes, however only when MyoVa FTNeu-eGFP was simultaneously expressed with pMEp12 plasmids, p12I/p8I localization showed to be altered from dots dispersed all over cytoplasm and cell surface to form cytoplasmic aggregates independently on variant of p12I expressed, suggesting that myosin Va plays an important role on traffics of p8I from Golgi to cell surface.

